# One size does not fit all: inter- and intraspecific variation in the swimming performance of contrasting freshwater fish

**DOI:** 10.1093/conphys/coaa126

**Published:** 2020-12-30

**Authors:** Peter E Jones, Jon C Svendsen, Luca Börger, Toby Champneys, Sofia Consuegra, Joshua A H Jones, Carlos Garcia de Leaniz

**Affiliations:** 1 Centre for Sustainable Aquatic Research (CSAR), Department of Biosciences, Swansea University, Singleton Campus, Swansea SA2 8PP, UK; 2 National Institute of Aquatic Resources (DTU-Aqua), Technical University of Denmark, Kemitorvet, Building 201, DK-2800 Kongens Lyngby, Denmark

**Keywords:** barrier passage, fish pass, metabolism, morphology, respirometry, selective effects

## Abstract

Artificial barriers cause widespread impacts on freshwater fish. Swimming performance is often used as the key metric in assessing fishes’ responses to river barriers. However, barrier mitigation is generally based on the swimming ability of salmonids and other strong swimmers because knowledge of swimming ability for most other freshwater fish is poor. Also, fish pass designs tend to adopt a ‘one size fits all’ approach because little is known about population or individual variability in swimming performance. Here, we assessed interspecific and intraspecific differences in the sustained swimming speed (*U_sus_*) of five freshwater fish with contrasting body sizes, morphologies and swimming modes: topmouth gudgeon, European minnow, stone loach, bullhead and brown trout. Significant *U*_sus_ variation was identified at three organizational levels: species, populations and individual. Interspecific differences in *U_sus_* were as large as 64 cm s^−1^, upstream populations of brown trout showed mean *U*_sus_ 27 cm s^−1^ higher than downstream populations, and species exhibited high individual variation (e.g. *cv* = 62% in European minnow). Sustained swimming speed (*U*_sus_) increased significantly with body size in topmouth gudgeon, European minnow and brown trout, but not in the two benthic species, bullhead and stone loach. Aerobic scope had a significant positive effect on *U*_sus_ in European minnow, stone loach and brown trout. Sustained swimming speed (*U*_sus_) decreased with relative pectoral fin length in European minnow and brown trout, whereas body fineness was the best predictor in stone loach and bullhead. Hence, swimming performance correlated with a diverse range of traits that are rarely considered when predicting fish passage. Our study highlights the dangers of using species’ average swimming speeds and illustrates why a ‘one size fits all’ approach often fails to mitigate for barrier effects. We call for an evidence-based approach to barrier mitigation, one that recognizes natural variability at multiple hierarchical levels.

## Teaser

This study reports substantial variation in the swimming ability of river-resident fish at three hierarchical levels: species, population and individual. Differences in swimming ability were related to a range of morphological and physiological traits. The results highlight the dangers of using average swimming speeds as a basis for barrier mitigation.

## Introduction

Artificial barriers such as dams, weirs and culverts are ubiquitous in rivers worldwide (Lehner et al., 2011; Januchowski-Hartley *et al.*, 2013; Grill *et al*., 2019; Belletti *et al.*, 2020) and cause numerous impacts on freshwater fish populations, including habitat fragmentation (Morita and Yamamoto, 2002; Santucci Jr *et al*., 2005), disrupted migrations (Lucas and Baras, 2008) and reduced connectivity (Wofford *et al*., 2005), which can make populations more vulnerable to other anthropogenic pressures (Fagan, 2002). To mitigate barrier impacts on fish, natural resource managers should identify those that are causing the most severe impacts (Kemp and O’Hanley, 2010). Passage through velocity barriers, such as culverts and sloping ramps, is highly dependent on fish swimming speed (Haro *et al.*, 2004; Peake, 2004; Castro-Santos, 2005; Castro-Santos, 2006; Weibel and Peter, 2013). Swimming performance data are also critical for the design of effective fish passes to provide passage over vertical barriers such as dams and weirs (Katopodis, 1992; Clay, 1995).

There has been historical bias in fish passage research, which has tended to focus on large, commercially important salmonids (Clay, 1995; Roscoe and Hinch, 2010). Crucially, salmonids are the ‘elite athletes’ of river fish communities (Webb, 1975), well known for their high swimming speeds and jumping ability (Stuart, 1962), and hence represent an exception, rather than a fair characterization of the wider river fish community (Birnie-Gauvin *et al*., 2019). A lack of swimming performance data for most non-salmonid species is likely to be one of the underlying reasons why salmonids appear to be three times more likely to pass the average fish pass (Noonan *et al.*, 2012). There has also been a tendency to focus on diadromous species when considering barrier effects, while river-resident taxa have largely been ignored (Lucas and Batley, 1996). This is perhaps due to the misconception that species that complete their lifecycles in rivers are sedentary and their longitudinal movements are negligible (Gerking, 1959). However, it is increasingly recognized that river-resident species regularly undertake long-distance movements for spawning (e.g. Lucas and Batley, 1996) and foraging (e.g. Schoby and Keeley, 2011) and these movements are also important to maintain gene flow between populations (Wofford *et al*., 2005). Hence, river-resident fish are also impacted by barriers, and knowledge of these species’ swimming abilities is crucial for predicting barrier effects, as well as identifying effective mitigation options (Kemp and O’Hanley, 2010).

Barriers (both natural and artificial) can affect colonization by invasive species (e.g. Townsend and Crowl, 1991; Vitule *et al*., 2012; Robinson *et al*., 2019), and in some cases, selective barriers have been used as a management tool (Rahel and McLaughlin, 2018). Consequently, there is often a trade-off between preventing the spread of invasive species and ensuring population connectivity of native species. Where invasive species are present, effective barrier management therefore requires detailed knowledge of the swimming ability of invasive species, as well as native taxa.

Fish passage guidelines tend to prescribe maximum flow velocities and barrier heights that should not be exceeded, and these are deemed suitable for broad groups of fish. For example, the UK Environment Agency fish pass guidelines suggest maximum flow velocities of 1.4–2.0 m s^−1^, and differential heads of 0.1–0.2 m, in a pool pass to ensure passage of ‘coarse fish’ (any freshwater fish other than salmonids; Armstrong *et al*., 2010). Such broad generalizations ignore potential variability in swimming performance, both at inter- and intraspecific levels (Taylor and McPhail, 1985; Tudorache *et al*., 2008).

River fish communities consist of species with different body shapes, physiological traits and swimming modes that define their realized niches (Willis *et al*., 2005; Poff and Allan, 1995; Montaña *et al.*, 2014; Pang *et al*., 2020). Additionally, individuals of the same species can show substantial trait variation at the population level due to adaptation to local environmental conditions (e.g. Taylor and McPhail, 1985; Pakkasmaa and Piironen, 2001; Webster *et al*., 2011). Riverine habitats show predictable longitudinal changes (Vannote *et al*., 1980), with headwater streams tending to be more turbulent and fast flowing, while lower catchment reaches tend to provide more slow-flowing habitat. These conditions should select for higher swimming ability in upstream headwater populations compared to downstream lowland populations. Even similar-sized individuals from the same population can vary 2-fold in swimming speed (Ojanguren and Braña, 2003), as well as differing markedly in functionally relevant morphological (Boily and Magnan, 2002) and physiological traits (Metcalfe *et al.*, 2016).

Here, we examined the extent of the interspecific and intraspecific variation in swimming performance of five species belonging to four contrasting families: two cyprinids (topmouth gudgeon *Pseudorasbora parva* and European minnow *Phoxinus phoxinus*), one nemacheilid loach (stone loach *Barbatula barbatula*), one cottid (bullhead *Cottus gobio*) and one salmonid (brown trout *Salmo trutta*). These species were chosen because they occupy contrasting habitat types (Maitland and Linsell, 2006), vary widely in body size and shape, and differ in swimming mode. Moreover, topmouth gudgeon, brown trout and European minnow have established invasive populations outside their native ranges, often with severe ecological impacts (Pinder *et al*., 2005; Museth *et al*., 2007; Jones and Closs, 2018), and swimming performance data for these species are important for invasive species management (Rahel and McLaughlin, 2018).

## Materials and methods

### Study species

Between 26 and 36 individuals of each species were collected by electric fishing (HT-2000 backpack machine, Halltech Aquatic Inc., Ontario, Canada) from populations in rivers and lakes in Wales (Table S1) in summer 2017 when water temperatures were between 15°C and 18°C. Topmouth gudgeon, European minnow, stone loach and bullhead were each collected from a single population, whereas brown trout was collected from two distinct catchments, each sampled from an upstream headwater and a downstream lowland site, to assess population-level variability in swimming performance. Brown trout was chosen for the population level study because they occur in a wide range of fluvial habitat types, ranging from small headwater streams to large slow-flowing rivers. Upstream sites were high elevation, steeply sloping, second-order streams characterized by turbulent fast flow, while downstream sites were lower catchment, low gradient, fifth-order rivers that offered more slow-flowing habitat (Table S1).

Fish were housed in separate 200 l cylindrical tanks in a 2500 l recirculating aquaculture system (TMC System 5000P, Tropical Marine Centre Ltd, Hertfordshire, UK). Individuals were marked using unique combinations of visual implant elastomer tags (Northwest Marine Technology, Anacortes, USA) and left to acclimatize for at least two weeks before swimming tests. Housing water temperature was maintained at 15 ± 1°C and photoperiod was set to 12 h:12 h light/dark cycle. Fish were fed daily (9 am) to satiation on pellet food (Atlantic Gold, Pacific Trading Aquaculture Ltd, Dublin, Ireland), supplemented with live maggots and frozen bloodworm.

### Swimming performance and metabolism

Swimming performance and metabolic rate (MR) were measured in one of four different sized swim tunnel respirometers (Loligo Systems, Viborg, Denmark), three Blaska-type tunnels and one Steffensen-type swim tunnel (Table S2; Fig. S1). The use of different tunnels ensured a suitable fish volume:water volume ratio for accurate measurement of MR (Svendsen *et al*., 2016). We followed best practice recommendations for allocating fish to different tunnels according to body weight (www.loligosystems.com; Table S2). Because fish size to tunnel size was kept as constant as possible, we are confident that potential side wall effects on swimming were kept to a minimum. Water velocities for each tunnel were carefully calibrated either using a purpose-built AC10000 flow meter (Loligo Systems, Viborg, Denmark) or using a proven dye tracing technique (Poulsen *et al*., 2012). Swimming speeds were also corrected for solid blocking effects (the increase in water velocity surrounding the fish caused by the fish body blocking a portion of the tunnel) following standard methodologies (Bell and Terhune, 1970). Water temperature was maintained at 15 ± 0.1°C in ambient water tanks using a temperature control set (Model AC10150; Loligo Systems, Viborg, Denmark). Air stones in ambient tanks ensured dissolved oxygen was always near saturation (>95%). Weekly cleaning of equipment and UV treatment of water ensured that bacterial respiration (measured at the end of each experiment) was negligible.

Test fish were weighed (±0.1 g) and measured for total body length (BL, mm) and maximum body girth (MBG, mm; see Fig. S2), before being introduced individually into the respirometers at 5 pm daily. AutoResp software (Loligo Systems, Viborg, Denmark) was used to automate the flush (180 s), wait (60 s) and measurement periods (420 s). Preliminary trials indicated that this flushing rate was sufficient to ensure dissolved oxygen never fell below 80%, and measurement periods were long enough to ensure an *R*^2^ > 0.9 for accurate measurement of O_2_ consumption (Genz *et al.*, 2013). Flow velocities were set to 1 cm s^−1^ to ensure adequate mixing of test water, and fish were left to acclimatize overnight. Oxygen partial pressure (kPa) in the test chambers was measured using fibre optic sensors (OX11250; Loligo Systems, Denmark) and mass-specific oxygen consumption rates (MO_2_; mgO_2_ kg^−1^ h^−1^) were calculated for each measurement phase using AutoResp software. Mass-specific oxygen consumption rates were used as a proxy for MR (Norin and Malte, 2011; Svendsen *et al*., 2013). Standard MR (SMR) was recorded at 9 am the following morning, calculated as the mean of the 10 lowest MR values during the 16 h test period (Norin and Malte, 2011).

Immediately after measurement of SMR, velocity was incrementally increased to measure maximum MR (MMR) and sustained swimming speed (*U*_sus_). Sustained swimming speed (*U*_sus_) is a measure of the aerobic swimming ability of fish (Brett, 1965), shows individual repeatability (Oufiero and Garland Jr, 2009) and is one of the most widely used metrics used in fish pass design (e.g. Clough *et al*., 2004; Laborde *et al*., 2016). The upstream half of the swim tunnels was covered to encourage a rheotactic response against the current (Fig. S1). Test velocities started at 5 cm s^−1^ and were increased in 5 cm s^−1^ increments every 9 min, while measuring *MO*_2_ (180 s flush, 60 s wait, 300 s measure), until fish stopped swimming effectively against the current. For the species that predominantly swam higher in the water column (topmouth gudgeon, European minnow and to a lesser extent brown trout), *U*_sus_ was defined as the point at which fish switched from a steady to an unsteady locomotory gait (Drucker, 1996). This point, known as ‘gait transition speed’, is recognizable in a range of fish species and is a reliable point at which to measure MMR and *U*_sus_ (Peake, 2008). Gait transition was not appropriate to measure *U*_sus_ in bullhead and stone loach because preliminary trials indicated that they did not show consistent active swimming but rather tended to use their pectoral fins and occasional tail beats to hold a benthic position at the upstream end of the swim tunnels. For these two species, *U*_sus_ was recorded at the point at which fish failed to maintain position at the upstream end of the chamber for over 10 s. Fish were observed constantly during swimming trials to identify the endpoints described above. MMR was estimated as the highest MO_2_ recorded (over a full 300 s measurement period), and aerobic scope (AS) was calculated as MMR minus SMR (Metcalfe *et al.*, 2016).

### Morphology

After testing in the respirometer, fish were euthanized via an overdose of 2-phenoxyethanol (following Home Office Schedule 1 procedures) and standardized photos (dorsal and lateral views) were taken using an overhead camera (Panasonic Lumix G2). Total BL, MBG, pectoral fin length (PL), caudal fin height (CL) and caudal fin area (CA) were measured (±1 mm; Fig. S2) using ImageJ (Schneider *et al*., 2012). Three metrics of body morphology were calculated due to their relevance for swimming ability (Fig. S2). Aspect ratio (AR) is a metric derived from the height and surface area of the caudal fin, and individuals with higher AR generally show higher swimming performance (Sambilay, 1990). Fineness ratio (FR) is a measure of how streamlined fish are, and more streamlined individuals tend to show higher swimming performance (Baktoft *et al*., 2016). Pectoral fin length ratio (PFLR) is a measure of pectoral fin length relative to BL, and individuals with longer pectoral fins tend to show higher swimming performance (Ojanguren and Braña, 2003).

### Statistical analysis

Interspecific differences in *U*_sus_ were tested by ‘ANCOVA’, with *U*_sus_ as the response variable, and ‘Species’ as the predictor, while statistically controlling for the effect of BL. Slope comparisons were examined using the ‘emtrends’ function in R package ‘emmeans’ (Lenth, 2019) to calculate Bonferroni corrections for multiple pairwise comparisons. Intercept comparisons were carried out using the ‘emmeans’ function in the same package. Interspecific differences in physiological and morphological traits were evaluated by general linear models with SMR, AS, MMR, BL, FR, PFLR and AR as the response variables and ‘Species’ as the explanatory variable. Trait values were log or square root transformed to stabilize variances and normalize residuals, where necessary.

Interspecific differences in the relationship between MR and swimming speed were explored using a linear mixed-effects model (LMM), with MR as the response variable, ‘Swimming speed’ and ‘Species’ and their interaction as fixed factors, and individual ‘FishID’ as a random factor to account for multiple measurements (at different swimming speeds) at the individual level. Pairwise comparisons of slope and intercept were carried out using the ‘emtrends’ and ‘emmeans’ functions.

Inter-population differences in *U*_sus_, SMR, MMR, AS, BL, FR, PFLR and AR in brown trout were examined using separate LMMs, with ‘Location’ (i.e. upstream or downstream) and BL as fixed effects and ‘Catchment’ as a random factor. The ‘lmerTest’ package (Kuznetsova *et al*., 2017) was used to estimate the statistical significance of model coefficients using the Satterthwaite’s approximation to calculate degrees of freedom.

Relationships between individual *U*_sus_ and traits were assessed using separate LMs for each species, fitting *U*_sus_ as the response variable and traits (BL, SMR, AS, FR, PFLR and AR) as explanatory variables. Model selection was undertaken using the ‘dredge’ function in the R package ‘MuMIn’ (Barton, 2018) to identify the most parsimonious model by minimizing corrected Akaike Information Criteria (AICc). Where more than one candidate model had similar levels of support (∆AICc < 2), the ‘model.avg’ function in ‘MuMIn’ was used to calculate parameter estimates across the ‘top model set’ (Grueber *et al*., 2011). All statistics were carried out using R statistical software (Version 3.6.1; R Core Team, 2019).

## Results

### Interspecific variation

**Figure 1:**
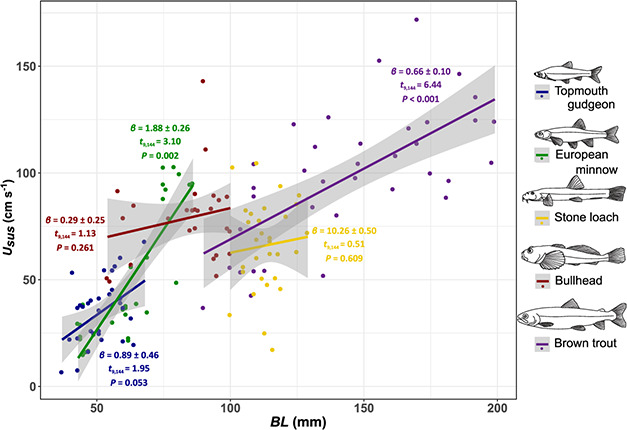
Interspecific variation in sustained swimming speed (*U_sus_*) with BL; slope estimates (*β*), *t* values and *P* values provided for each species.

**Figure 2:**
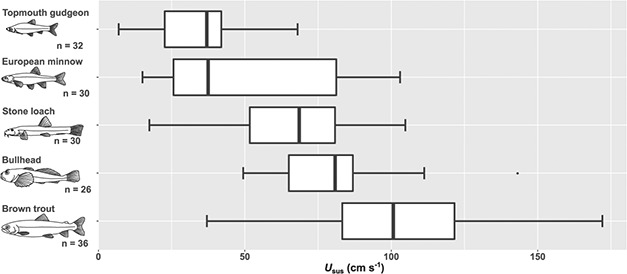
Interspecific differences in sustained swimming speed (*U*_sus_).

Sustained swimming ability showed significant interspecific differences (*F*_4,144_ = 53.97, *P* < 0.001), when the effect of BL (*F*_1,144_ = 74.50, *P* < 0.001) was accounted for (Fig. 1), and there was a significant interaction between ‘Species’ and BL (*F*_4,144_ = 5.84, *P* < 0.001). Mean *U*_sus_ ranged from a minimum of 35 ± 5 cm s^−1^ in topmouth gudgeon to 99 ± 10 cm s^−1^ in brown trout (mean ± 95% confidence interval (CI); Fig. 2). European minnow showed a significantly higher slope than all other species (pairwise differences: ∆*β* ≥ 1.22, t.ratio_144_ ≥ 2.86, *P* < 0.039), except topmouth gudgeon (pairwise difference: ∆*β* = 0.99 ± 0.53, t.ratio_144_ = 1.87, *P* = 0.336). No other interspecific differences in slope were statistically significant (*β* ≤ 0.63, *t*_4,144_ ≤ 1.32, *P* ≥ 0.679). Controlling for the effect of *BL*, two pairwise species comparisons were significant: European minnow showed significantly higher *U*_sus_ than both stone loach (*α* = 46 ± 14, t.ratio_144_ = 3.31, *P* = 0.010) and trout (*α* = 43 ± 11, t.ratio_144_ = 3.98, *P* = 0.001). There was little indication that interspecific variation in *U*_sus_ was related to any of the other traits measured (Table 1).

**Table 1:** Interspecific differences in physiological and morphological traits and *U*_sus_ for comparison (mean ± SE).

	Topmouth gudgeon 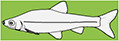 *n* = 32	European minnow 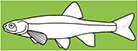 *n* = 30	Stone loach 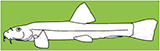 *n* = 30	Bullhead 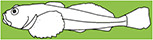 *n* = 26	Brown trout 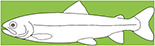 *n* = 36
**Physiological trait**					
SMR (mgO_2_ kg^−1^ h^−1^)	138 ± 7_A_	199 ± 13_B_	109 ± 5_C_	108 ± 5_C_	122 ± 3_A_
MMR (mgO_2_ kg^−1^ h^−1^)	473 ± 27_A_	712 ± 37_B_	403 ± 21_C_	360 ± 11_C_	534 ± 12_D_
AS (mgO_2_ kg^−1^ h^−1^)	334 ± 24_A_	512 ± 41_B_	294 ± 20_AC_	252 ± 13_C_	411 ± 12_D_
**Morphological trait**					
BL (mm)	53 ± 1_A_	64 ± 3_B_	116 ± 1_C_	86 ± 3_D_	147 ± 6_E_
FR (ratio)	0.210 ± 0.003_AD_	0.204 ± 0.003_AB_	0.155 ± 0.005_C_	0.216 ± 0.007_D_	0.197 ± 0.003_B_
PFLR (ratio)	0.137 ± 0.004_A_	0.174 ± 0.003_B_	0.153 ± 0.003_C_	0.247 ± 0.007_D_	0.176 ± 0.003_B_
AR (ratio)	2.65 ± 0.10_A_	2.34 ± 0.12_B_	1.28 ± 0.04_C_	1.19 ± 0.06_C_	2.10 ± 0.04_B_

SMR, standard metabolic rate; MMR, maximum metabolic rate; AS, aerobic scope; BL, body length; FR, fineness ratio; PFLR, pectoral fin length ratio; AR, aspect ratio; significant differences are denoted by different letters.

**Figure 3:**
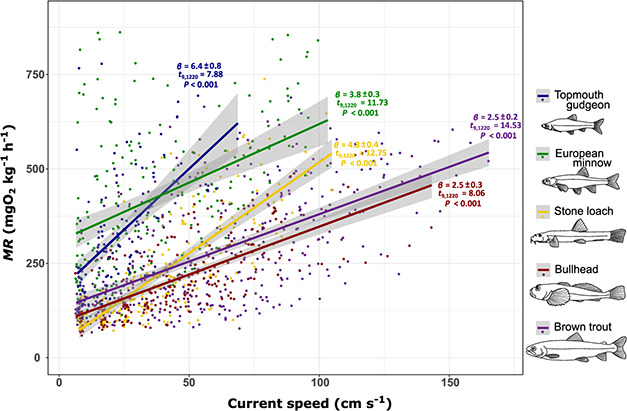
Interspecific variation in MR with current speed in swimming respirometers; slope estimates, *t* values and *P* values for each species.

There were significant interspecific differences in the relationship between MR and swimming speed (*F*_9,1220_ = 128.6, *P* < 0.001; Fig. 3). European minnow and topmouth gudgeon showed substantially higher mass-specific MRs (typically 200–300 mgO_2_ kg^−1^ h^−1^) at low swimming speeds (<20 cm s^−1^) compared to stone loach, bullhead and brown trout (typically 100–150 mgO_2_ kg^−1^ h^−1^). Bullhead and brown trout showed significantly lower slopes in the relationship between MR and swimming speed compared to European minnow and topmouth gudgeon (t.ratio_173–215_ > −4.01, *P* < 0.001). These differences in swimming energetics were largely in line with behavioural observations during swimming trials: European minnow and topmouth gudgeon actively swam even at very low current speeds, whereas bullhead, brown trout and stone loach tended to maintain position using their pectoral fins at lower current speeds (<50 cm s^−1^), generally only swimming actively at current speeds exceeding 50 cm s^−1^.

### Population variation in brown trout

**Figure 4:**
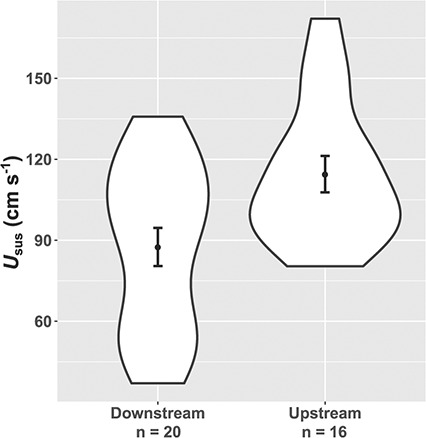
Differences in sustained swimming speed (*U*_sus_) between brown trout from upstream headwater and downstream lowland populations (mean ± SE).

Brown trout from upstream populations showed significantly higher *U*_sus_ (mean ± standard error (SE) = 115 ± 7 cm s^−1^) than those from downstream populations (mean ± SE = 88 ±7 cm s^−1^; Fig. 4), when the effects of BL and ‘Catchment’ were controlled for (*t*_32_ = 2.97, *α* = 21.84 ± 7.35, *P* = 0.006). No upstream–downstream population trait differences were observed, except for upstream populations of brown trout showing significantly lower *PFLR* (*t*_34_ = −2.36, *b* = −0.007 ± 0.003, *P* = 0.024; Table S3).

### Individual variation

There was substantial intraspecific variation in *U*_sus_ (Fig. 2), with bullhead varying the least (*cv* = 25%) and European minnow the most (*cv* = 62%). Sustained swimming speed (*U*_sus_) increased significantly with *BL* in topmouth gudgeon, European minnow and brown trout, but not in bullhead or stone loach (Table 2). AS showed a significant positive relationship with *U*_sus_ in European minnow, stone loach and brown trout, but not in bullhead or topmouth gudgeon. Sustained swimming speed (*U*_sus_) increased significantly with *FR* only in stone loach (*P* = 0.014).

**Table 2:** Model averaged parameter estimates for best performing models (∆AICc <  2) predicting relationship between intraspecific variation in *U*_sus_ and the various morphological and physiological traits examined

Species	Trait	*β ±* SE	*z* or (*t*) value	*P* value
Topmouth gudgeon (*n* = 32) 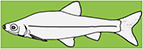	BL AS	0.90 ± 0.32 0.02 ± 0.02	2.67 0.45	0.008 0.655
European minnow (*n* = 30) 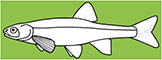	BL AS PFLR	1.74 ± 0.20 0.04 ± 0.01 −454 ± 165	(8.77) (3.05) (−2.75)	<0.001 0.005 0.011
Stoneloach (*n* = 30) 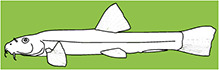	AS FR SMR	0.09 ± 0.03 315 ± 122 0.14 ± 0.14	2.78 2.47 1.00	0.005 0.014 0.316
Bullhead (*n* = 26) 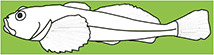	FR AR BL	226 ± 122 19 ± 13 0.29 ± 0.26	1.77 1.40 1.06	0.077 0.160 0.288
Trout (*n* = 36) 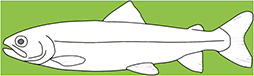	AS BL PFLR AR SMR	0.14 ± 0.05 0.66 ± 0.12 −924 ± 426 −22 ± 13 −0.27 ± 0.18	2.57 5.15 2.09 1.61 1.43	0.010 <0.001 0.037 0.106 0.151

BL, body length; SMR, standard metabolic rate; AS, aerobic scope; FR, fineness ratio; PFLR, pectoral fin length ratio; AR, aspect ratio.

## Discussion

Our study reveals that a ‘one size fits all’ approach for estimating fish swimming performance in relation to barrier passability is not tenable. Substantial interspecific variation in swimming ability was observed, with mean *U*_sus_ differing by as much as 64 cm s^−1^ even among species of similar body size. As barrier impacts are related to swimming ability (Castro-Santos, 2006; Makrakis *et al*., 2007; Castro-Santos and Haro, 2010), our study indicates considerable potential for velocity barriers to select against weak swimmers. Moreover, as the interspecific differences in *U*_sus_ were strongly influenced by body size, barriers may have size-selective effects (Volpato *et al*., 2009; Noonan *et al*., 2012).

Barriers associated with road crossings (e.g. culverts) tend to be abundant in river systems globally (Januchowski-Hartley *et al.*, 2013; Mantel *et al.*, 2017; Jones *et al.*, 2019; Belletti *et al.*, 2020), so, based on our data, selective effects based on swimming ability and body size are likely to be widespread. Passage of culverts by weaker swimming fish can be facilitated by adding baffles (Newbold *et al.*, 2014), and decreasing baffle spacing can improve passage of small-bodied fish (Cabonce *et al.*, 2019). Our results also serve to highlight the challenge of designing efficient fish passes for diverse groups of fish. Fish pass hydraulics should be designed with flow velocities low enough to accommodate the weakest-swimming target fish. However, faster-swimming fish can sometimes be deterred from entering fish passes with insufficient attraction flows (Williams *et al*., 2012). In this sense, fish passes that provide diverse flow conditions (e.g. nature-like fishways) are likely to be most successful in allowing passage of groups of fish with contrasting swimming abilities (Bunt *et al*., 2012; Williams *et al*., 2012).

MR increased rapidly with flow velocity in most species, suggesting that even swimming at speeds considerably lower than *U*_sus_ requires substantial energetic expenditure, which has considerable implications for predicting barrier effects on fish movement. For example, while passage of a single instream structure might be well within the maximum swimming speed of fish, the presence of multiple structures will likely have a cumulative effect that may be beyond their energetic scope (Armstrong *et al*., 2010; Roscoe and Hinch, 2010). Additionally, the energetic cost of passage may leave fish with insufficient energy reserves to reproduce or complete other basic life history functions (Caudill *et al*., 2007; Thiem *et al*., 2016). There are also clear implications for the provision of resting pools in fish pass design, which are added in an effort to prevent fatigue (Katopodis, 1992; Castro-Santos and Haro, 2010; Williams *et al*., 2012). If flow speeds within resting pools are not sufficiently low, fish may be unable to negotiate other parts of the fish pass (Castro-Santos and Haro, 2010). Our study indicates that more benthic-swimming species (bullhead, stone loach and to a lesser extent brown trout) were able to maintain position in low flow velocities (5–15 cm s^−1^) with relative ease (close to SMR values) by holding position using pectoral fins, while the more pelagic species (European minnow and topmouth gudgeon) had to spend substantially more energy by active swimming. Thus, flow velocities in resting pools may need to be lower for some pelagic-swimming species than for benthic fish.

Velocity barriers rarely present uniform flow conditions, and fish passes generally offer resting places with slow flows, pinch points where maximum flows are found and a range of flow speeds between these extremes (Katopodis, 1992; Clay, 1995). The Environment Agency (UK) fish pass guidelines (Armstrong *et al.*, 2010) suggest maximum flows of 1.1 m s^−1^ in culverts to allow passage of course fish and less than 1.25 m s^−1^ to allow passage of brown trout. These values were higher than the *U*_sus_ of 98% of course fish and 86% of brown trout in our study. Prescribed flow speeds for pool passes are also higher than the *U*_sus_ of the vast majority of fish in our study (1.4–2.0 m s^−1^ for coarse fish and 1.7–2.4 m s^−1^ for brown trout; Armstrong *et al.*, 2010). Fish use a combination of anaerobic burst (at pinch points), sustained (moderate velocity areas) and endurance (in rest areas) swimming types to negotiate obstacles (Castro-Santos, 2006) so the guideline flow speeds would not necessarily prevent passage. However, our data do suggest that even culverts and fish passes built to best practice guidelines are likely to be energetically demanding for many fish and a large proportion of fish are likely to be excluded from upstream passage. The poor performance of fish passes globally (Noonan *et al.*, 2012) is likely to be at least in part due to overestimation of swimming performance and underestimation of the energetic demands of passage. Some options for improving passage efficiency include reducing flow speeds, increasing rest areas and limiting the number of pinch points where energetically demanding burst swimming is required.

The significantly higher *U*_sus_ observed in upstream populations of brown trout compared to downstream populations is consistent with *a priori* predictions, based on higher flow velocities in headwater areas selecting for higher swimming ability (Taylor and McPhail, 1985; Páez *et al*., 2008; Leavy and Bonner, 2009). Brown trout can inhabit a much wider range of hydrological conditions than that covered by our study (Lobón-Cerviá and Sanz, 2017) so it is likely that population-level variation may be much greater than observed here. There was no evidence that the observed population-level differences in *U*_sus_ were due to body size, but individuals from the upstream populations had shorter pectoral fins relative to their body size, which has previously been associated with higher swimming ability (Rouleau *et al*., 2010). The upstream–downstream population differences could be due to local adaptation (Garcia de Leaniz *et al*., 2007) or phenotypic plasticity (Oufiero and Whitlow, 2016). Irrespective of the drivers, the results indicate river managers also need to take population location into account when considering barrier effects and mitigation options.

The extent of intraspecific variation in *U*_sus_ was unexpected (e.g. 37–172 cm s^−1^ in brown trout) and highlights the importance of working with the full range of swimming abilities that species exhibit, rather than using mean values. To effectively mitigate barrier impacts, fish passes should aim to provide passage for all individuals (Baras and Lucas, 2001), but using mean swimming speeds as benchmarks would inevitably select against the weakest-swimming individuals. This highlights the need to explicitly consider potential selective pressures of barriers and fish passes on fish communities (e.g. Volpato *et al*., 2009).

At the intraspecific level, *U*_sus_ showed a positive association with BL in European minnow, brown trout and topmouth gudgeon. In contrast, *U*_sus_ was unrelated to BL in stone loach and bullhead, perhaps indicating other traits are more important in benthic species. The positive relationship identified between *U*_sus_ and AS in European minnow, stone loach and brown trout is consistent with other studies (Reidy *et al*., 2000; Killen *et al*., 2012) and shows the importance of considering metabolism in fish passage. The negative relationships we observed between *U_sus_* and PFLR in brown trout and European minnow was unexpected as longer pectoral fins have been previously shown to confer better station-holding ability and faster swimming speeds (Arnold *et al*., 1991; Ojanguren and Braña, 2003). However, our findings are in agreement with Rouleau *et al*. (2010) who found salmonids with shorter pectoral fins swam faster, possibly because short fins reduce drag. Overall, our results indicate that the drivers of intraspecific variation in swimming speed vary between species and are more complex than simple size-related variation.

The potential use of velocity barriers to prevent passage of invasive fish has been put forward by several studies (Newbold *et al.*, 2016; Rahel and McLaughlin, 2018; Zielinski *et al.*, 2019). Dispersal along river catchments is a major pathway for secondary invasions in topmouth gudgeon (Pinder *et al.*, 2005), but the wide range of *U*_sus_ observed emphasizes the difficulties in designing effective selective barriers to prohibit their passage. The maximum *U*_sus_ of topmouth gudgeon was 68 cm s^−1^, which was above the mean *U*_sus_ for many of the native taxa. To be effective, selective barriers need to prevent all invasive individuals passing, without disrupting the passage of native species. In this case, using a threshold of > 68 cm s^−1^ to prevent passage of topmouth gudgeon would clearly impair the passage of native species. Hence, the use of velocity barriers in controlling invasive fish will often be challenging, needs to be carefully considered, and requires detailed knowledge of the full range of swimming performance of both invasive and native species.

We used four different swim tunnels to test *U*_sus_ in a range of fish sizes to ensure accurate measurement of MRs. While we followed best practice to minimize any potential tunnel size effect (e.g. keeping fish volume:water volume relatively consistent, carefully calibrating current velocities, and correcting for solid blocking effects) we cannot be absolutely sure that the use of different tunnels did not affect swimming behaviour. Unfortunately, controlling for any such effect statistically was not possible as tunnel size was completely confounded by fish body size so this approach would have led to erroneous conclusions. Ultimately, we are confident that we used the best possible approach to simultaneously test *U*_sus_ and measure MRs across a range of fish sizes.

Longitudinal migrations have been documented in river-resident brown trout (Clapp *et al.*, 1990), bullhead (Knaepkens *et al.*, 2005), European minnow (Nunn *et al.*, 2010) and stone loach (Maerten *et al.*, 2007). These movements are crucial for spawning, foraging, accessing refugia, counteracting downstream displacements in high flows and allowing recolonization of vacant habitat patches following disturbance (Lucas and Baras, 2008). Even where such movements are rare, they are very important to support gene flow between populations (Junker *et al.*, 2012). Free movement is therefore essential to the maintenance of healthy river fish communities, but velocity barriers and ineffective fish passes are disrupting these movements. It is crucial that river managers worldwide base decisions on representative swimming data for the whole target fish community.

## Conclusions

Our study shows substantial variability in *U*_sus_ among species, among populations and among individuals within populations. Swimming speed is a major determinant of passage success (Haro *et al*., 2004; Castro-Santos, 2006) and migration rates (Eliason *et al*., 2011). There is a general consensus that traditional methods in fish pass design are failing (Noonan *et al*., 2012; Kemp, 2016; Birnie-Gauvin *et al*., 2019), and new approaches are needed. There is a need to move away from a ‘one size fits all’ approach to address natural variability in swimming performance within river fish communities. Barrier removal should always be considered, but in cases where removal is not feasible, we suggest that fish passes affording diverse and spatially heterogeneous flows (e.g. nature-like fish passes; Katopodis *et al*., 2001; Calles and Greenberg, 2005) offer the option that best embraces the variability in swimming performance existing in natural populations.

## Funding

This study was funded by the EC Horizon 2020 Research & Innovation Programme (AMBER Project, grant agreement No. 689682).

## Supplementary Material

Supplementary_material_coaa126
