# Supplementary material for: One size does not fit all: inter- and intraspecific variation in the swimming performance of contrasting freshwater fish
Source: Conserv Physiol. 2020 Dec 30;8(1):coaa126. doi: 10.1093/conphys/coaa126 (PMC7772615; doi:10.1093/conphys/coaa126)
Supplement: Supplementary_material_coaa126 [file supplementary_material_coaa126.docx]

**SUPPLEMENTARY MATERIAL**

**Table S1** Collection locations for fish species used in experiments. D/S indicates a downstream location within a catchment, whereas U/S indicates an upstream location

| **Species** | **Collection site** | **n** | **Body length (mm)** | **Coordinates** | **Wetted width (m)** | **Elevation (m)** | **Slope**  **(°)** | **Stream order** |
| --- | --- | --- | --- | --- | --- | --- | --- | --- |
| Topmouth gudgeon | Lleidi Reservoir | 32 | 37-68 | 51.716622 N, -4.14063 E | NA | 80 | NA | NA |
| European minnow | River Tawe (D/S) | 30 | 43-86 | 51.724527 N,-3.84124 E | 15 | 30 | 0.2 | 5 |
| Stone loach | River Neath (D/S) | 30 | 100-121 | 51.6958 N,-3.732398 E | 18 | 20 | 0.1 | 5 |
| Bullhead | River Tawe (U/S) | 26 | 54-100 | 51.724527 N,-3.84124 E | 4 | 360 | 2.3 | 2 |
| Brown trout | River Tawe (U/S)  River Tawe (D/S)  River Afan (U/S)  River Afan (D/S) | 8  8  7  13 | 189-186  90-182  140-198  100-199 | 51.724527 N,-3.84124 E  51.724527 N,-3.84124 E  51.682334 N,-3.619514 E  51.603376 N, -3.776048 E | 4  15  4  12 | 360  30  240  20 | 2.3  0.2  2.0  0.4 | 2  5  2  5 |

**Table S2** Specifications of swim tunnels used for swimming respirometry experiments

| **Model** | **Volume**  **(L)** | **Length**  **(cm)** | **Internal diameter/width (cm)** | **Cross sectional area**  **(cm^2^)** | **Flow velocity**  **(cm s^-1^)** | **Fish weight range**  **(g)** |
| --- | --- | --- | --- | --- | --- | --- |
| SW10000 | 0.17 | 10 | 2.64 | 5.47 | 1-50 | 1-4 |
| SW10030 | 1.7 | 20 | 5.5 | 22.9 | 1-100 | 4.1-12 |
| SW10030 | 2.5 | 25 | 5.5 | 22.9 | 1-100 | 12.1-40 |
| SW10100 | 10 | 40 | 10 | 100 | 1-140 | >40 |

**Table S3** Differences in physiological and morphological traits between upstream and downstream populations of brown trout (mean ± SE). *SMR* – standard metabolic rate, *MMR* – maximum metabolic rate, *AS* – aerobic scope, *BL* – body length, *FR* – fineness ratio, *PFLR* – pectoral fin length ratio, *AR* – aspect ratio. Significant differences are denoted by different letters (*t* > 1.96, *P* < 0.05).

| **Trait** | **Upstream** | **Downstream** |
| --- | --- | --- |
| **Physiological** |  |  |
| SMR (mgO_2_ kg^-1^ h^-1^) | 125 ± 6_A_ | 121 ± 4_A_ |
| MMR (mgO_2_ kg^-1^ h^-1^) | 532 ± 20_A_ | 537 ± 15_A_ |
| AS (mgO_2_ kg^-1^ h^-1^) | 407 ± 20_A_ | 415 ± 15_A_ |
| **Morphological** |  |  |
| BL (mm) | 147 ± 7_A_ | 143 ± 8_A_ |
| FR (ratio) | 0.193 ± 0.004_A_ | 0.200 ± 0.003_A_ |
| PFLR (ratio) | 0.172 ± 0.002_A_ | 0.178 ± 0.002_B_ |
| AR (ratio) | 2.05 ± 0.06_A_ | 2.14 ± 0.06_A_ |


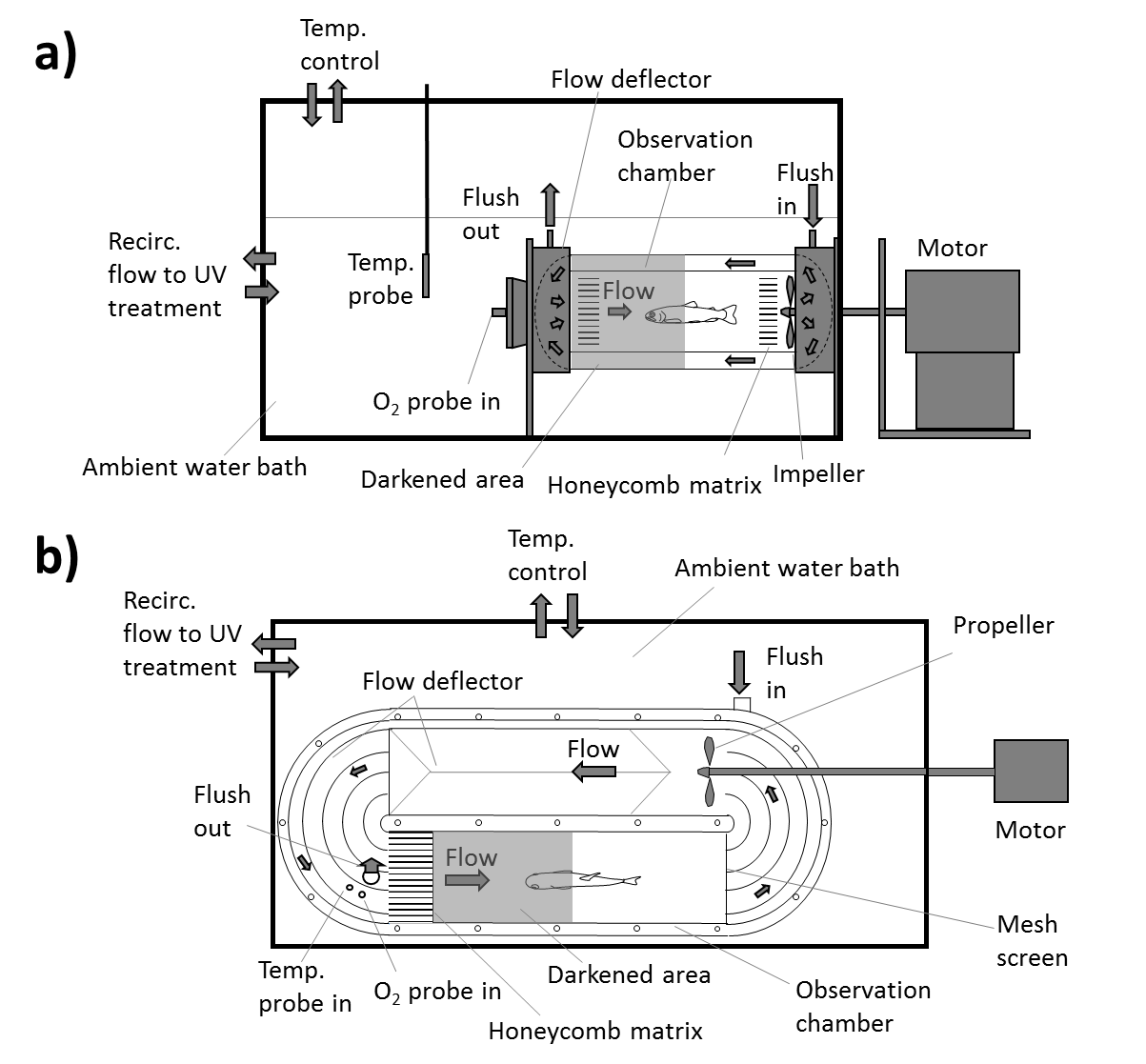


**Figure S1** - a) lateral view of the 0.166, 1.698 and 2.450 L modified Blaska-type swim tunnel respirometers, b) aerial view of the 10 L Steffensen-type swim tunnel respirometer. The 0.17L, 1.5L and 2.0L swim tunnels all had cylindrical observation sections, and impellers located at the downstream end of observation chambers were driven by electric motors that generated a recirculating flow. Deflectors collimated flow and honey comb matrices (3mm diameter) at either end of the test chambers created approximately laminar (micro-turbulent) flow conditions within the test area. The 10L chamber had a cuboid observation chamber and a propeller produced an approximately laminar, micro-turbulent, recirculating flow, utilising collimators and honey-comb matrices. Black curtains around the respirometers minimised disturbance of test individuals by the experimenter. Flow velocities within the 10L swim tunnel were calibrated using a handheld digital flow meter, while water speeds in the other tunnels were calibrated by repeatedly video tracking movement of dye through the observation chamber and correlating with motor voltage.


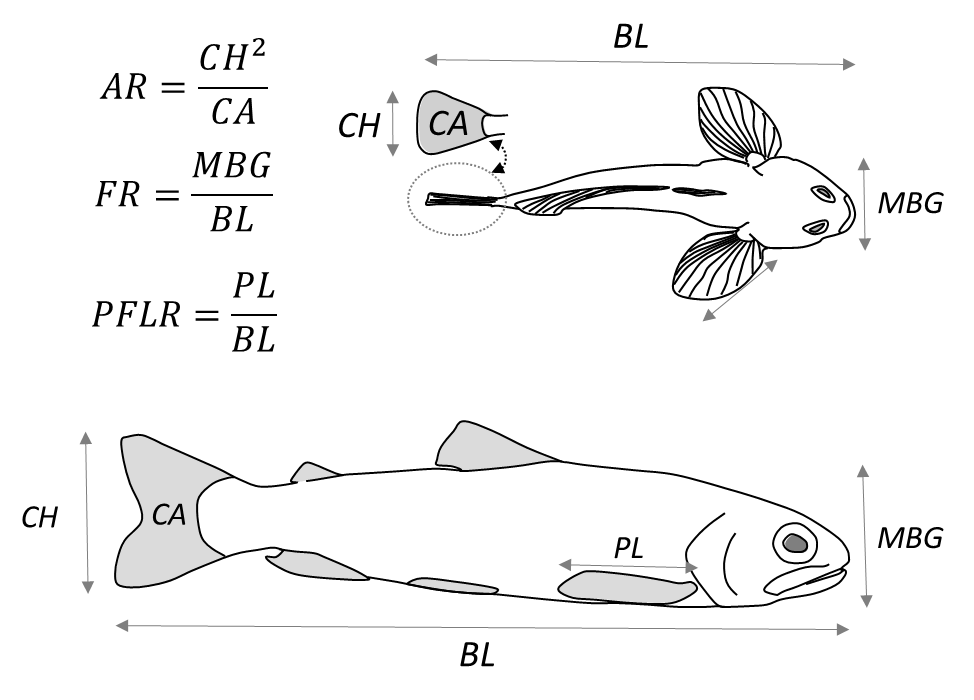


**Figure S2** Morphometrics collected for each fish. *AR* = aspect ratio, *CH* = caudal fin height, *CA* = caudal fin area, *FR* = fineness ratio, *MBG* = maximum body girth, *BL* = body length, *PFLR* = pectoral fin length ratio, *PL* = pectoral fin length.
